# Editorial: Molecular mechanisms and immunotherapeutic targets in nanomedicine-based cancer therapy

**DOI:** 10.3389/fonc.2024.1468397

**Published:** 2024-09-27

**Authors:** Rabeea Siddique, Ghulam Nabi, Suliman Khan

**Affiliations:** ^1^ Department of Cerebrovascular Diseases, The Second Affiliated Hospital of Zhengzhou University, Zhenzhou, China; ^2^ First Affiliated Hospital of Xinxiang Medical University, Xinxiang, China; ^3^ Department of Zoology, Institute of Molecular Biology and Biotechnology, University of Lahore, Lahore, Pakistan

**Keywords:** nanomedicine, immunotherapeutic, cancer, immune response, nanomaterial

In today’s world, cancer remains a significant threat, making the pursuit of effective treatments even more critical (1). It is a major public health, societal, and economic problem of the 21^st^century, causing almost one in six deaths and one in four deaths from noncommunicable diseases worldwide (1). Despite worldwide high fatality, efficacy of available treatment options including surgery and chemotherapy is limited (2–4). Although traditional therapies are crucial to managing cancer, their effectiveness is limited by numerous challenges including poor drug solubility, drug resistance, and difficulty targeting tumors (5). Using nanomedicine signals a new era characterized by greater specificity, efficacy, tolerability, and improved drug cellular uptake in cancer therapy (5).

In this Research Topic, we have focused on cancer therapy using nanomedicines to develop advanced diagnostic and therapeutic strategies. Researchers and clinicians are actively contributing to developing techniques for rapid detection and efficient treatment of various cancer types, as well as to further elucidate the molecular and immunological regulations in cancers. Waheed et al. in this Research Topic, highlighted the importance of lipid-based nanoparticles (LBNPs) in cancer therapy, suggesting biocompatibility and biodegradability of the LBNPs. The development of novel lipids and enhanced LBNP formulations could open up new possibilities for the use of LBNPs in drug delivery. Si et al. indicated the importance of engineered exosome versus natural exosome as a vehicle in cancer therapy. Engineered exosomes can be modified in a variety of ways to enhance or even confer new properties according to the needs. Moreover, Jia et al. discussed the role of m6A modification in the prognosis and drug resistance of gastric cancer patients with HER2-positive cells. A variety of modulators for m6A (such as methyltransferases) and FGFR4 can be targeted for different therapeutic options, including immunotherapy and chemotherapy.

Zhao et al. investigated the prognostic significance of KIT exon 11 mutation subtypes in patients with GISTs. Overall, 233 patients were diagnosed with gastrointestinal stromal tumors (GISTs) followed by analyzing clinicopathological characteristics and prognosis among the different mutation subtypes. Point mutations were detected frequently by somatic mutational followed by compound mutations and tandem duplication mutations. According to the results presented, point mutations depicted lower mitotic count with a high recurrence rate. On the other hand, deletions and compound mutations depicted high mitotic count and intermediate risk of recurrence. This study further showed that multi-variation analysis high recurrence risk groups had worse prognostic values in specified tumors. Overall, this study indicated that mutations in exon 11 of the KIT gene are common with high or intermediate recurrence risk in patients with GISTs. Pelorca et al. have reported that Paget’s disease of the breast (PDB) is classified as clinical and subclinical/pathological in the presence of areolar abnormalities and in all other cases respectively. In this study, 85 patients were included with clinical PDB of 58.8%, where 27.1% were detected with stage 0 and 92.9% had multicentric disease. In addition, most of the patients (83.5%) were presented with HER2 or luminal HER2 molecular subtype, where patients with clinical PDB showed higher rate of *in situ* disease. Overall, the authors concluded PDB as a rare condition associated with HER2 overexpression as well as multifocality/multicentricity.

Cancer is a leading public health problem of the 21^st^ century, causing high mortality and financial constraints. Developing advanced diagnostic tools can help in the early diagnosis of cancer, which can help in better management and treatment. Furthermore, using advanced engineered molecular targets can eliminate cancer cells with more specificity and efficiency.
